# From impact metrics and open science to communicating research: Journalists’ awareness of academic controversies

**DOI:** 10.1371/journal.pone.0309274

**Published:** 2025-01-27

**Authors:** Alice Fleerackers, Laura L. Moorhead, Juan Pablo Alperin, Michelle Riedlinger, Lauren A. Maggio

**Affiliations:** 1 School of Journalism, Writing, and Media, University of British Columbia, Vancouver, British Columbia, Canada; 2 School of Publishing, Simon Fraser University, Vancouver, British Columbia, Canada; 3 Department of Journalism, San Francisco State University, San Francisco, California, United Sates of America; 4 School of Communication, Queensland University of Technology, Brisbane City, Queensland, Australia; 5 College of Medicine, University of Illinois Chicago, Chicago, IL, United States of America; Dental Hypothesis, ISLAMIC REPUBLIC OF IRAN

## Abstract

This study sheds light on how journalists respond to evolving debates within academia around topics including research integrity, improper use of metrics to measure research quality and impact, and the risks and benefits of the open science movement. It does so through a codebook thematic analysis of semi-structured interviews with 19 health and science journalists from the Global North. We find that journalists’ perceptions of these academic controversies vary widely, with some displaying a highly critical and nuanced understanding and others presenting a more limited awareness. Those with a more in-depth understanding report closely scrutinizing the research they report, carefully vetting the study design, methodology, and analyses. Those with a more limited awareness are more trusting of the peer review system as a quality control system and more willing to rely on researchers when determining what research to report on and how to vet and frame it. While some of these perceptions and practices may support high-quality media coverage of science, others have the potential to compromise journalists’ ability to serve the public interest. Results provide some of the first insights into the nature and potential implications of journalists’ internalization of the logics of science.

## Introduction

The systems of scholarly communication and journalism are deeply intertwined, with changes in one influencing the values, norms, and practices of the other [1, 2]. Yet, the mechanisms underpinning these interconnections are not well understood, with a lack of evidence into how journalists make sense of, and adapt their work in response to, changes in scholarly communication [3]. To help fill this gap, we present a qualitative analysis of 19 interviews with health and science journalists from the Global North aimed at understanding their perceptions of key scholarly communication debates (e.g., on “impact” metrics, research integrity, open science [OS]) and how these perceptions, in turn, relate to the professional norms, practices, and criteria they use to select, verify, and communicate research.

### Evolving debates within scholarly communication

In recent years, changes in the scholarly communication ecosystem have sparked debates about interrelated concerns regarding research integrity, challenges of assessing research quality, and adverse effects of impact metrics, as well as on the potential role of OS to either address or exacerbate these concerns. These concerns, and the debates around them, are complex, involving actors at every level of scientific systems, especially researchers and administrators at academic institutions.

Underscoring them all is a “publish-or-perish” culture that exerts pressure on academics to produce as many research articles as possible and ensure they are published in prestigious and “high impact” journals [4, 5]. This pressure has led to a hyper-competitive academic environment that relies heavily on mechanisms for tracking and assessing research and researchers for resource allocation (e.g., career progression, departmental funding, etc.) [6]. The effects of these practices are system-wide and form part of a larger set of changes that some have described as the neoliberalization of the university [7, 8]. Fully addressing the nuances of these discussions is beyond the scope of this literature review, but we touch on key concerns in the following paragraphs, as they pertain to how scholars understand and talk about their work with each other and, potentially, with the journalists they engage with.

### Concerns about research integrity

On a pragmatic level, the growing pressures to publish have fueled multiple concerns about research integrity. Early among these was the so-called “replication crisis” stemming from the high volume of studies whose findings could not be verified, either due to lack of methodological detail or accessible data or because results were not robust enough [9]. More recently, the rise of a pay-to-publish model fueled the rise of so-called “predatory journals” that charge authors but do not offer a legitimate peer review process or follow other editorial best practices [10, 11]. Today, one of the most pressing concerns is the emergence of “paper mills” that sell co-authorships in real, or sometimes machine-written, articles that authors can add to their CVs [12, 13]. These specific concerns and the resulting debates have led to multiple responses from the academic community, including greater calls for research assessment reform [14], close scrutiny of the peer review system [15], and efforts towards greater openness and transparency [16].

### Challenges in research assessment

One place to enact such changes in research assessment is in review, promotion, and tenure (RPT) processes, which often purport to reward many aspects of academic work but are known to place emphasis on the number of publications [17] and the conflated concepts of prestige, quality, and impact [18]. Proposed alternatives include the use of narrative CVs (i.e., which allow those being assessed to describe their career trajectory) [19], development of values-based indicators [20], and other assessment frameworks that place emphasis on the quality over quantity of research outputs [21].

Generally, calls for research assessment reform have focused on pushing back on the simplistic use of citation-based metrics, especially the Journal Impact Factor (JIF) [14, 22]. While there is broad consensus on the limitations and adverse effects of the JIF [23], its use for research assessment remains widespread [4], in part because of the labour of assessing the quality of a growing number of publications. Eve and Priego [24] argue that relying on these indicators is driven by a need to conserve reading labour: the metrics let scholars decide what, in the vast body of literature, is worth reading, and who among their peers is worth rewarding.

Peer review is another way in which the scholarly community has traditionally delineated between problematic and reliable or “authoritative” scholarship [25]. Yet, like journal metrics, the effectiveness of peer review itself has been subject to debates in recent years. These debates largely centre on the (in)effectiveness and (in)appropriateness of peer review for weeding out problematic research, but also include other considerations such as biases in the review system and their impact on the diversity of the scholarly record; the burden of peer review on already strained academics; and the lack of transparency involved [15, 26–28].

### Benefits and risks of open science

These debates about research integrity, impact, and assessment have been further framed by ongoing discussions about OS. The concept of OS is itself debated, with some focusing closely on the opportunities for greater access and transparency in the scientific process, and others emphasizing the opportunities it brings for engaging more broadly with other societal actors [29, 30]. The closest thing to a consensus definition of OS is the one offered by the unanimously adopted UNESCO Recommendation on Open Science [31]:

an inclusive construct that combines various movements and practices aiming to make multilingual scientific knowledge openly available, accessible and reusable for everyone, to increase scientific collaborations and sharing of information for the benefits of science and society, and to open the processes of scientific knowledge creation, evaluation and communication to societal actors beyond the traditional scientific community (p. 7).

That is, in this broad sense, OS seeks to make research products and knowledge more inclusive and accessible, both to scientists and society more broadly. However, while debates in the Global North place emphasis on the value of OS for scientists, debates in the Global South, especially in Latin America, place greater emphasis on the interactions with non-academic actors, particularly through citizen science and science communication [29, 30, 32].

In the debates dominated by the Global North, where the journalists we interviewed were based, proponents of OS argue that practices such as sharing research data and protocols or publishing open peer review reports can bring transparency to the research and publication process and, in turn, improve the integrity of the scholarly record [33], shift the scholarly community’s reward system toward valuing a more diverse range of research outputs [34], or more broadly help to realign incentives as part of the push for research assessment reform [35].

Advocates also see OS as a mechanism for making scholarly communication more equitable and inclusive, as openness can allow less well-resourced scholars to access and, for some forms of OS, contribute to research knowledge [31, 36]. Yet others have raised concerns that some forms of OS may compound existing inequities in whose research is published and rewarded [30, 37]. For example, the reliance on article processing charges to publish open access (OA) in some journals are an additional barrier to publishing in a system that is already laden with barriers and structurally disadvantages scholars from less-resourced institutions and countries. Moreover, some fear that the growing use of un-peer-reviewed preprints in scholarly communication‚ as was seen during the COVID-19 pandemic, could contribute to the spread of flawed science, misinformation, and even conspiracy theories [38, 39].

### Close relationships and heavy reliance between science and the media

In reporting on research, journalists must thus navigate this contentious and rapidly evolving scholarly communication landscape and ongoing questioning of how research should be vetted, valued, and rewarded. A growing body of theoretical and empirical work suggests that journalists’ close relationships with scientists may enable these challenging debates to influence how journalists think about and cover research, shaping the nature of the media coverage that reaches the public.

Decades of research have demonstrated the close and codependent relationships that can develop between science journalists and the researchers whose work they report on [40, 41]. These studies show that science journalists rely heavily on interviews with researchers to help them identify new story ideas, vet the quality of studies, contextualize and translate the findings for public audiences, frame the implications, and more [42]. Journalists sometimes also rely on researchers for access to research papers that are behind paywalls [43, 44]. Researchers, in turn, rely on journalists to share new findings to the public, gain societal legitimacy, and win funding—a reliance that may be increasing as scientists and scientific institutions face growing pressures to demonstrate a wider societal “impact” of their work [45, 46].

Journalism and academia share many commonalities—including a shared commitment to independent, rigorous investigation in pursuit of “truth”—but they are also distinct in important ways, including differing epistemologies, ethical frameworks, and standards of practice [47]. As such, although the close relationships between journalists and scientists may enable them to work together more efficiently [48], they also come with risks. For journalists, internalizing norms, perspectives, or values of science may result in media coverage that prioritizes the interests of researchers over those of the public. Moreover, journalists’ heavy reliance on scientists could prevent them from reporting what is relevant to society (i.e., rather than what is relevant for particular scientists or scientific institutions). It could also compromise their ability to provide a balanced portrait of reality that gives space for competing interpretations or dissenting perspectives, or to act as watchdogs of the powerful (e.g., scientists, funders, pharmaceutical companies) [49].

Understanding the potential risks and benefits of journalists’ proximity to scientists is especially important given the evolving debates discussed above [50]. For instance, a nuanced understanding of problematic aspects of science (e.g., research fraud, retractions, improper use of metrics) is essential if journalists are to sound an alarm when the actions of scientists put the public interest at risk. Yet, a more incomplete understanding of these issues could result in media coverage that inaccurately portrays research infractions of evidence that science is “broken,” damaging public trust [51, 52].

Similarly, journalists’ awareness of OS practices—such as the use of preprints, OA papers, or open data sets—may facilitate their work by enabling them to use research knowledge that would otherwise be inaccessible [53–55]. Yet, a more superficial understanding of these practices—or one that prioritizes the interests of science over those of the public—could result in problematic media coverage, such as stories conflating the OA movement with predatory publishing [56, 57] or stories that present preprints as if they were peer reviewed research [58–60].

### Theoretical framework: The scientization of journalism

This study explores journalists’ response to these interconnected controversies using a theoretical framework grounded in the concept *medialization of science*, defined as the “increasingly tighter coupling of science and the mass media” [40] that can develop as scientists and journalists come to depend on one another [61]. While medialization is most often used to consider how scientists bend towards the norms, values, practices, or *logics* of journalism, it is theoretically bidirectional and could also be used to understand how journalists adopt the logics of scientists [62]. In this study, we focus on this second direction of medialization—i.e., the *scientization* of journalism. The concept of scientization has proven useful for describing environmental journalists’ increasing reliance on scientific and expert sources [63], as well as “the influence that researchers exert on the media as they vie for their research (or opinions) to be featured in lieu of other coverage” [3]. However, it remains understudied [3].

Theoretically, scientization could lead journalists specializing in science topics (e.g., health, science, environment) to be especially susceptible to internalizing scientific logics because of their frequent interactions with, and dependence on, researchers and their research. Over time, such interactions may encourage journalists to internalize perspectives of the scientists they interview. These perspectives may, in turn, shape journalists’ professional practices for reporting on science. We explore this possibility by addressing the following research question:

*RQ*: *How and to what extent do journalists who report on science perceive key scholarly communication debates (e*.*g*., *on “impact” metrics*, *research integrity*, *OS) and how do these perceptions inform their professional journalistic logics (i*.*e*., *the norms*, *practices*, *and criteria they use to select*, *verify*, *and communicate research)*?

## Methods

This research was guided by a constructivist paradigm, in which meaning is conceptualized as created through a “reciprocal and emergent” process of individual and collective interpretation [64]. In line with this view, we conducted an interview study using qualitative description [65], meaning that we aimed to gather data that describe the “why, how, and what questions about human behavior, motives, views, and barriers” [66]. We selected this methodology as it is well suited for use when interviewing individuals with direct experience of the studied phenomenon. Moreover, such an exploratory, qualitative approach was appropriate given the lack of research on the scientization of journalism.

This study is a component of a larger research project in which we conducted interviews with journalists and scientists about their perceptions and experiences relating to media coverage of science. We have previously published other findings from this project about journalists’ use of preprints [67] and the medialization of scientists [61]. Given our current research aim, the present study focuses only on the journalists’ interviews, drawing on those sections of the transcripts that relate to the academic debates described above. The Simon Fraser University Research Ethics Board (# 30000244) and the San Francisco State Institutional Review Board (#2021175) exempted the project from further review.

**Fig 1:**
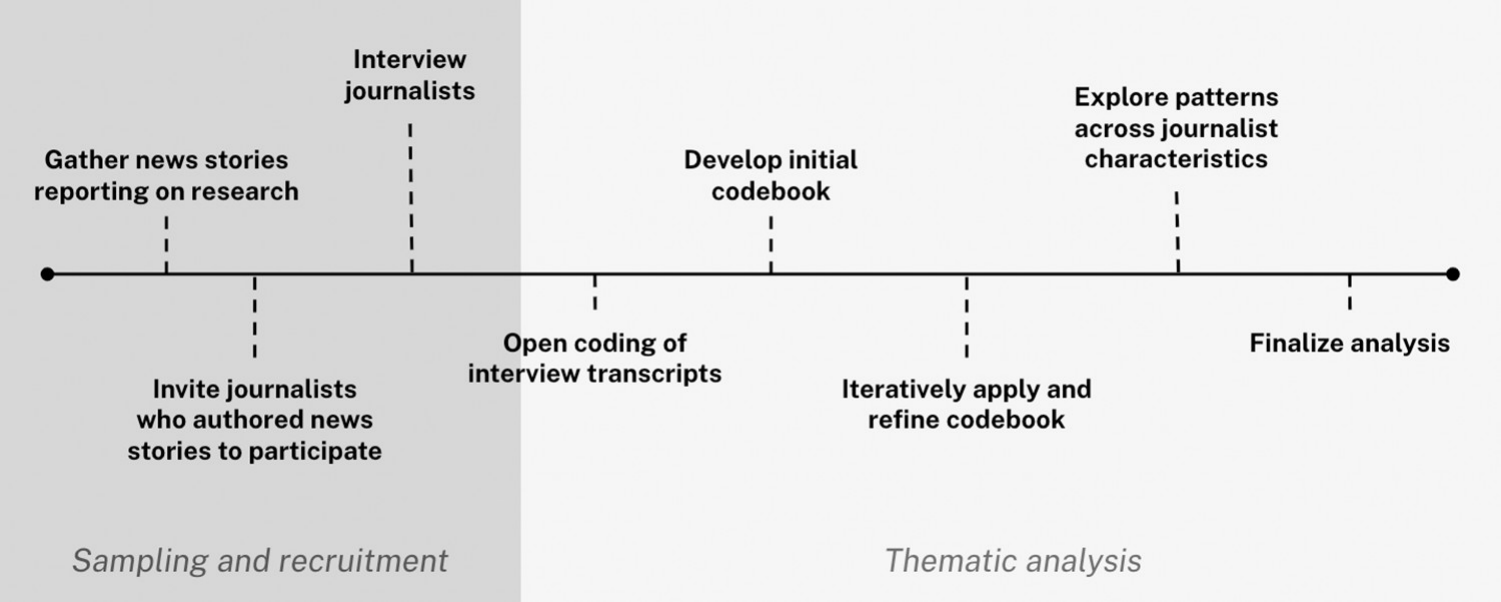
Overview of study methodology.

Our methodology is summarized in Fig 1 and described in detail below.

### Sample

Eligible participants for this study worked for one of seven news outlets that included a mixture of traditional, legacy news organizations (i.e., *The Guardian*, *New York Times*); historically print-only science magazines (*Popular Science*, *Wired*); digital-native health sites (*News Medical*, *MedPage Today*); and a science blog (*IFLScience*). We selected these news outlets based on their focus on science and health news and frequent coverage of academic research [68], and because their diversity (in formats, publishing models, audiences) is more representative of today’s digital media ecosystem than a sample of traditional, legacy news outlets [69]. Journalists from these outlets were eligible to participate if they had published a story between March 1 and April 30, 2021, that included a mention of research. Mentions of research were identified through the outlet’s RSS feed or via the Twitter timeline of the official account that posted a link to every story. We have made the scripts used for this process openly available [70].

### Data collection

Between July and November 2021, a research assistant with experience in journalism emailed 121 eligible journalists to participate using publicly available contact information. We stopped recruiting journalists when the research team agreed we had reached *data sufficiency*—the point at which the interview data were both in-depth and broad enough to allow us to achieve our research objective [71]. Based on this criteria, we interviewed 19 journalists from the Global North (see Table 1 for an overview of participant characteristics). Journalists consented to participate in the study via a consent form sent before the interviews were conducted.

**Table 1:** Characteristics of journalists who participated in interviews (n = 19).

Journalist	Primary outlet	Primary outlet description	Employment status (staff or freelance)	Years in journalism
**J1**	IFLScience (“I fucking love science” IFLS)	UK-based science blog	On staff	6
**J2**	Popular Science	US-based science news and feature publication	Freelance	7
**J3**	Popular Science	US-based science news and feature publication	Freelance	2
**J4**	Popular Science	US-based science news and feature publication	Freelance	6
**J5**	Wired	US-based science, technology and culture publication	Freelance	33
**J6**	Medpage Today	US-based medical news service provider	On staff	1
**J7**	Medpage Today	US-based medical news service provider	Freelance	25
**J8**	Popular Science	US-based science news and feature publication	Freelance	1–2
**J9**	Wired	US-based science, technology and culture publication	On staff	4
**J10**	IFLScience	UK-based science blog	On staff	8
**J11**	Popular Science	US-based science news and feature publication	On staff	25
**J12**	Medpage Today	US-based medical news service provider	On staff	6
**J13**	The Guardian	UK-based news and media publication	Freelance	10
**J14**	The Guardian	UK-based news and media publication	On staff	14
**J15**	Wired / Ars Technica	US-based science, technology and culture publication / US-based technology, science and political news publication	Freelance	8
**J16**	Wired	US-based science, technology and culture publication	On staff	28
**J17**	Popular Science	US-based science news and feature publication	On staff	7
**J18**	New York Times	US-based daily news publication	Freelance	9
**J19**	The Guardian	UK-based news and media publication	On staff	3

NB. To protect journalist identities, education information is reported in aggregate only. All 19 participants had received at least one educational certificate or degree, with all but 2 reporting that they had a bachelor’s degree or higher in a social sciences and humanities (SSH) field (n = 17). Many journalists also had training in a Science, Technology, Engineering, or Math (STEM) field (n = 6), with 3 journalists stating that they had attained a graduate degree in this area. Finally, 8 participants reported having received professional journalism education through a certificate, bachelor’s, or master’s program. Table first published in Fleerackers et al. [67].

The research assistant conducted and recorded semi-structured interviews with the journalists via Zoom. In the interviews, participants were asked to describe their professional experience reporting on research, including how they engage with scientists. The interview guide included questions about journalists’ background and experience, professional practices and motivations for using research, and perceptions of preprints and press officers [72]. Questions about key academic debates were not included in the interview guide but were often mentioned by participants organically when responding to other questions, prompting us to explore these emergent themes in more detail through the present study. Interviews were on average 35 minutes (range: 10–47 minutes). A third-party transcriptions service transcribed all transcripts, which were then de-identified and spot checked by LLM prior to analysis. Participants were not provided the transcripts for review.

### Data analysis

Analysis of the data began with AF identifying and highlighting sections of the transcripts broadly applicable to the participants’ descriptions of their engagement with scientific debates and practices. All authors used these highlighted transcripts to conduct open coding of elements that might relate to journalists’ understanding and adoption of scholarly debates and norms.

The authors met virtually to discuss and co-create a working codebook, which was subsequently refined by AF after additional readings of the transcripts (i.e., codebook thematic analysis) [73]. The authors then independently applied the codebook to the transcripts, while staying open to the identification of additional codes, and again met to discuss areas requiring revision. Informed by these conversations, familiarity with the data, and literature on journalism and scholarly communication, AF iteratively refined and applied the newly organized codes to transcripts, and, eventually, solidified the codebook.

Given the different profiles of the journalists in our sample, we also explored potential patterns in how different types of journalists understood academic debates and how this affected their work. To do so, AF did a final round of coding using the qualitative analysis software NVivo 12 [74], which allows researchers to assess whether themes are expressed by all participants or by only a subset with particular characteristics.

Examining potential differences among types of journalists was important as some of the participants had professional training or education in science (and thus may be more aware of controversies in academia), while others did not. Similarly, some worked as specialized science journalists at science-focused outlets, whereas others reported on other beats (e.g., lifestyle, culture) or worked for other types of outlets (e.g., general news outlets). The specialized journalists could potentially be more sensitized to scholarly debates and norms than those with less experience reading academic research or interacting with scientists.

While our qualitative design did not allow us to quantitatively compare different groups of journalists, this final round of coding confirmed that the themes described in the “Results and interpretation of themes” section were expressed by journalists of different educational backgrounds, levels of education, years of experience, roles, and specializations, and by those working for specialized health/science outlets as well as generalist publications.

### Research team reflexivity

Our research team included individuals with a variety of backgrounds and experiences as researchers (all authors), journalists (AF and LLM), and communication professionals (AF and MR) who have worked in North American contexts. JPA’s research focuses on scholarly communication in Latin American and globally, while LAM studies the prevalence of irresponsible research practices by scientists. Additionally, all members of the research team shared a background and interest in OS and the responsible use of research metrics. Taken together, the research team’s interests and expertise likely influenced the study aims, analysis, and conclusions.

## Results and interpretation of themes

Our results suggest that journalists’ professional logics are influenced by debates within academia about research integrity, impact metrics, and OS. Yet, journalists varied widely in terms of how closely they were enmeshed with these debates, with some presenting a highly critical and nuanced understanding and others presenting a more limited awareness. These different levels of awareness contribute to different approaches to reporting on research. We report these varied perspectives and practices below and discuss their implications for science media coverage. As context, we provide direct quotes from participants across our sample. Participants are identified by their participant number (e.g., J11 is journalist participant 11).

### Critical awareness of academic debates

Some journalists had a deep awareness of ongoing debates and discourses taking place within the scholarly community—an awareness that sometimes influenced how they selected, verified, and communicated research. For example, journalists expressed a nuanced understanding of the limitations of peer review, including its slow, potentially biased, and often imperfect effectiveness as a quality-control mechanism [15, 75]. Others referenced challenges to research integrity, such as the ongoing replication crisis, rise of predatory journals, pressure to publish and its implications for research quality, and problematic nature of publication bias. J11, for instance, noted that “there are certain—what’s the word—certain pressures, you know, to sort of publish or perish and that people might be kind of cranking out a lot of articles.”

### Skepticism and scrutiny of research quality

This awareness of ongoing challenges to the integrity of science led some journalists to approach findings with additional scrutiny. This was the case for J1, whose knowledge of the replication crisis made them skeptical of using older research if “there’s no follow-up” or newer studies that have come to similar conclusions. Such skepticism contrasts with prior research finding that journalists often uncritically accept perspectives and findings from scientists [76, 77].

Perhaps because of their awareness of research integrity issues, journalists described relying on scientific methods for ensuring the research they reported was accurate, unbiased, and trustworthy. This included, above all, a consideration of the study design and methodology. Several journalists noted that they lacked the expertise to verify complex statistical analyses, as has been described in prior research [78]. However, they still scrutinized the quality of the research, something notoriously difficult to do even within the scientific community [79].

Common strategies employed by journalists included critically reading the paper and investigating the nature and size of the sample. They valued human subjects above mice and considered larger samples more trustworthy than smaller ones. One journalist, however, noted that small samples are appropriate in qualitative studies, demonstrating a more nuanced understanding of research methods and study design. Meta-analyses and randomized controlled trials (RCTs) were preferred over other types of studies, which aligns with how researchers conceptualize the hierarchy of available evidence [80, 81].

Journalists looked for causality, in ways similar to scientists, asking questions such as, “Is there an implicit bias in the study? Are the conclusions valid? Is it really a causation, for example, or just a correlation?” [J12] and noting whether “their methodologies leave this open to be, like, a correlation, not necessarily a causation” [J15].

Finally, some journalists saw research as more credible when researchers noted study limitations and did not have any clear conflicts of interest, aligning with the consensus building in the scientific community that transparent reporting is an indicator of research integrity [82]. A few journalists went even further, running their own statistical tests on publicly available datasets to provide new insights. These journalists made comments such as, “I was very much playing the scientist, almost, in that I was the one writing, ‘What does the data mean?’” [J2].

This added scrutiny has important implications for the public, as it could help address decades-long calls for journalists to move beyond uncritical “science cheerleading” [83, 84], more thoroughly vet the research they report [85], and provide balanced, contextualized media coverage of science [86, 87]. It also aligns with calls among scholars to assess the quality of research itself rather than the reputation of the journal it is published in [24]. However, journalists’ skepticism about certain forms of research, such as those employing small samples or exploratory study designs, could further entrench existing biases, such as journalists’ disproportionate coverage of the health sciences and other traditionally quantitatively oriented fields [88, 89].

### Making research public—a double edged sword

Journalists’ critical understanding of academic debates extended beyond issues of research quality to debates about how, when, and to what extent the scholarly community should make research outputs public. For example, some journalists noted the increasing need for universities to establish legitimacy and gain public support through self-interested science public relations efforts, echoing similar findings to those described by Weingart [46]. This was reflected in comments such as “[Research papers] come to me either directly if I’ve worked with them before or through their press release offices, right, which are pumping out research all the time in order to garner interest” [J4]. Journalists’ knowledge of these promotional pressures sometimes resulted in a hesitance or, occasionally, distrust of public relations efforts, as has long been observed in prior research [90]. However, more recently, Comfort et al. found that public relations materials can indeed influence science journalists, with up to 65% of news article sentences displaying “high similarity” to press release material [91].

Journalists were not hesitant about all modes of making research knowledge public. When it came to the public’s right to access research—a core pillar of the OS movement [31]—journalists were much more positive overall. Their reflections on the public’s right to research knowledge were often grounded in journalists’ own frustrations in accessing the literature, as evidenced by J19: “it’s so frustrating when you finally find the paper that you want to read and it’s behind a paywall, particularly when it’s publicly funded research, you know?”

Yet journalists also discussed OS in other contexts, such as when reflecting on the rise of preprints during the COVID-19 pandemic. Journalists recognized the benefits that scientists find in preprints, both as a rapid-sharing mechanism that avoids the slow process of peer review and publication and as a way to circumvent paywalls [92]. As J16 explained,

[Preprints are] the way that scientists talk quickly to each other, and especially as people realize that they don’t have easy access to the big journals because they cost a lot of money and they’re behind paywalls, that this becomes much more of a way for scientists to talk to each other and to a potential audience for that work.

Others, such as J12, expressed awareness of the importance of establishing priority as a scientist to avoid getting “scooped.” Indeed, this “registration” function of scholarly communication has been widely touted as a benefit of preprints [93]. For J12, this knowledge, in turn, informed how and when they reported on preprint research:

…you have to cover [a] preprint as soon as possible…Give the researchers that put the preprint also another, how to say it, another stamp of—they were the first—that their research was the first, because sometimes, that can be problematic in scientific circles [J12].

While it is uncontroversial for preprints to serve this registration function, it is rather uncertain to what extent they can serve the “recognition” function traditionally served by peer-reviewed journals [93]. Journalists’ coverage of preprints, which saw a spike during the pandemic and has subsequently declined [94], may be unwittingly providing preprints a stamp of approval that reinforces the perspective that preprints can or should supplant peer-reviewed journals in academic incentive and reward systems.

Importantly, journalists’ relatively optimistic view of openness may be specific to the forms of OS that journalists focused on in their interviews—namely, OA articles and preprints. Both types of outputs provide access to complete research findings, rather than underlying data, methods, or materials [55]. That is, the journalists we interviewed, all of whom were from the Global North, seemed to have an understanding of OS that aligns with what de Oliveira et al. [30] describe as the “administrative view” that focuses on transparency and efficiency. As long as journalists focus on these aspects of OS, it seems unlikely that they will actively play a role in opening “the processes of scientific knowledge creation” to society, as envisioned by the UNESCO Recommendation on Open Science [31]. Instead, the opposite seems likely to be true. This type of limited engagement with OS seems more likely to contribute to an environment in which scientists’ views and expertise are given priority, whereas society is seen as the recipient of that expertise.

### Limits in understanding of academic debates

Journalists’ in-depth understanding of academic debates was not universal, however. Alongside journalists who were skeptical of peer review were journalists who saw it as the “gold standard” [J13] to guarantee that findings were trustworthy and credible enough to report on. Similarly, while some journalists were aware of the pressures researchers faced to demonstrate “impact,” others uncritically accepted measures of impact that are widely used, but heavily critiqued, within academia.

### Peer review, popularity, and prestige as proxies of research quality

Journalists’ relatively limited understanding of the nuances surrounding debates about research integrity translated to superficial and, at times, potentially problematic practices for vetting research. For instance, journalists relied heavily on peer review when deciding what research to cover, as seen in comments such as, “with the peer reviewed piece, you’re really saying ‘This is a discovery or something that is fairly well-vetted and legitimate’” [J18]. Scholars have noted that journalists use peer review as a stamp of approval, one which moves the findings from uncertain evidence to confirmed facts [41, 77, 95]. This view of peer review aligns with that of many researchers [96] despite criticisms that peer review is not a reliable quality control system and can, at times, introduce biases [15, 26]. However, this view may be doubly problematic when taken by journalists who then communicate to publics who may not have an understanding of the scientific process as cyclical and self-correcting [97].

In addition, journalists described selecting studies to report based on indicators such as the number of citations an article receives, the impact factor of the journal it is published in, and the reputation of the journal, the authors, and their institutions [5, 98]. This reliance on proxies for research quality and impact can be seen in comments such as “you can do stuff to verify … the impact factor of a journal or how legitimate the research is” [J9] and “that’s why they’re in *Science* and *Nature*, ‘cause they’re big stories, they’re important” [J5]. As J10 explained, over time, these indicators formed an internalized framework that journalists could fall back on without having to critically scrutinize study findings:

We’ve got, like, a fairly good understanding of we know the journals that you almost don’t have to question too much, you know, like, the *Nature* journals, *Science*, *PLOS ONE*, all the *PLOS* ones, you kind of realize, like, these are fairly well-respected, so you don’t have to question them too much.

The focus on reputational assessment strategies aligns with findings of Badenschier and Wormer [99] and Rosen et al. [100], who similarly identified “scientific relevance” as a value shaping science journalists’ selection decisions. Deferring decisions about the impact or quality of scientific outputs to recognized scientific community mechanisms potentially saves time for journalists, but our findings reveal how doing so can enable problematic aspects of academia to seep into journalism, such as an overreliance on citation-based metrics of “impact” and journal reputation [24, 56]. Citation indicators such as the JIF are known to be poor measures of the quality of individual articles and to be biased against journals in the social sciences and humanities, as well as those from the Global South [23, 101].

### Scientists and science promotion—an uncritical reliance

Similarly, while some journalists were aware that scientists faced pressures to promote their work, this did not translate to a critical relationship with press officers or the scientists whose work they promoted. Instead, journalists looked to journal and university press officers to understand the research, as reflected in comments such as:

I couldn’t do my job without the public information officers. They’re great, and especially for some of the studies that are written in a language that even I find quite difficult to access, like nothing is better than a press release to just get your head straight about what the top-line findings of something are. [J18]

Relatedly, none of the journalists in our sample mentioned the importance of maintaining independence from the scientists they interviewed, even though independence from sources is one of journalism’s core values [102] and a foundational ethical principle of science journalism [85]. Instead, journalists described their relationships to scientists as collaborative and trusting, as reflected in statements such as, “Obviously, I have my own sources, and if I have a source who’s in that topic area, I would reach out to them,” [J17] and, “I have sources that I rely on and that I think are trustworthy, and I go to them” [J7]. Journalists expected scientists to act as unbiased experts on the topic at hand, even while acknowledging their fallibility. As J7 noted, incorporating expert opinions “gives [the story] credibility that makes me feel better, even though, yes, they sometimes are not all that accurate” [J7]. Such reliance on previous relationships and more established scholars can contribute to the “Matthews Effect” in science [103] and goes counter to the equity goal at the heart of many academic controversies and debates.

As noted in previous research [41, 67], journalists leaned on these trusted sources to critique and vet other researchers’ work. Yet, journalists relied on scientists for more fundamental aspects of their reporting as well. Many asked experts to translate the research—to “give more insight and clear explanation to the article” [J1]. This was especially important for journalists without a background in science:

One thing is that, again, often kind of coming in as a liberal arts backgrounded person with not a huge understanding of something like computational fluid dynamics, that I would hope that they are a resource I can trust, you know, and, again, that gets into a tricky thing, because, you know, if I can’t tell that they’re wrong, you know, who’s going to tell me they’re wrong? [J11]

Journalists also used scientists when seeking out research to cover or incorporate in their stories, “relying on the expertise of people who research in that area to point me towards the like, external literature to begin with” [J14].

On a practical level, journalists also frequently used scientists to get access to (paywalled) research papers [44, 104]. This was true even of participants working at major publications, such as the staff reporter at *The Guardian* who reported that, “A lot of the time, it’s really hard to find a PDF of the paper, and, like, scientists are brilliant. If you email them, they send you a PDF of the paper” [J19]. In this sense, scientists not only acted as collaborators in journalists’ work [61] but also as gatekeepers and agenda setters. Their actions and input helped shape what research got coverage, as well as how it was contextualized and framed [105].

Science journalists’ overly reliant relationships on expert sources have been discussed elsewhere [85, 87] and align with the narrower definitions of scientization used in previous research—i.e., the increasing influence of scientific and expert sources on journalists’ work [3, 63]. Our findings extend this previous research and expand our understanding of scientization by providing a view into how and why journalists work with particular scientists and how they come to negotiate this intersection of seemingly disparate professions.

### Limitations

These findings must be considered in light of several limitations. First, the journalists we interviewed responded to the recruitment email, which suggests they likely had an existing interest in science relations and may thus have been more enmeshed in scholarly communication debates than others. They also represented a specific subset of journalists—those who reported on research at least occasionally, produced text-based stories, worked for online (rather than broadcast) media outlets, wrote in English, and were based in the Global North. The participants were also interviewed during the second year of the COVID-19 pandemic, when public interest in science was high [106] and concerns about research integrity were growing [107]. The practices and understanding of academic debates we identified may thus differ from those we might find among journalists working in other geographic, linguistic, professional, and temporal contexts.

The qualitative nature of the study design means that it was not possible to systematically assess differences among journalists based on publication outlets, reporting interests, or other characteristics. Future research could test our science debates and professional journalistic logics framework with a larger sample of science journalists and a study design that would allow for comparisons across participant contexts and characteristics.

## Discussion

In this study, we used in-depth interviews with journalists who report on science to further explore and understand the implications of recent debates in scholarly communication and the close relationships that can develop between journalists and scientists [40, 46, 61]. It revealed how evolving academic debates influence both journalists’ perception of research studies (e.g., as valuable or high-quality) and their professional logics (norms and practices for reporting on them). These findings have implications for the fields of science communication, journalism, and scholarly communication—an intersection of the fields in which all co-authors are firmly established—presenting some of the first evidence of the impacts of the “scientization” of journalists described by MacGregor et al. [3]. The findings contribute to scholarly understanding of the interrelationships between science and journalism and shed light on journalistic logics and associated practices with important practical implications.

In some instances, journalists possessed a nuanced understanding of academic debates about research integrity, which appeared to encourage a more critical approach to communicating science that could benefit their audiences. For example, some journalists expressed a deep awareness of the limitations of peer review and approached research studies with a skeptical eye. They decided which studies to report on by adopting practices used by many scholars, such as considering the study design and methodology and, in some cases, doing their own research (e.g., assessing the existing literature, collecting and analyzing their own data, and creating meaningful data visualizations).

At other times, however, journalists expressed a more superficial understanding of challenges facing academia, which sometimes translated to problematic practices for reporting on research. For instance, some journalists described relying on academic citation metrics or markers of prestige as proxies for research quality—both practices that are themselves critiqued within academia [23]. None of the journalists we interviewed appeared to be concerned about maintaining independence from the scientists they were covering in their work, and most also trusted the information they received from university and journal press officers. This uncritical reliance on scientific experts risks compromising their ability to serve the public interest and retain independence and autonomy from their sources [102, 108].

Finally, journalists—as seen in the quotation “Who’s going to tell me they’re wrong?”—often recognized their limited understanding and ability to vet research. Yet, their efforts to verify or triangulate research sometimes moved them closer to academia’s controversies and insular practices. Moving forward, there is a need to explore how journalists might better understand and document research integrity independently, without an overreliance on scientists.

Relatedly, our results suggest that advocates of OS have allies in science journalism, as journalists, too, appear to believe that research knowledge should be public knowledge. It is unclear, however, whether journalists see themselves as an integral part of the broader OS movement, in which they—and not just scientists—are responsible for advancing transparency and improving research integrity and for creating opportunities for public participation at all stages of the research process (e.g., by reporting on the increasing number of datasets, protocols, and other research outputs that are openly available [55]). As such, researchers may wish to further explore the relationship between professional journalistic logics and OS, including how open research outputs shape journalists’ practices for selecting, verifying, and communicating research to the public. Additionally, it would be valuable to examine how professional journalistic logics, and the practices they inform, are advancing the goals of opening up the processes of science to society [55].
